# The diagnostic and prognostic value of systems biology research in major traumatic and thermal injury: a review

**DOI:** 10.1186/s41038-016-0059-3

**Published:** 2016-09-21

**Authors:** Jon Hazeldine, Peter Hampson, Janet M. Lord

**Affiliations:** 1 NIHR Surgical Reconstruction and Microbiology Research Centre, Institute of Inflammation and Ageing, Birmingham University Medical School, Birmingham, B15 2TT UK; 2 Healing Foundation Centre for Burns Research, Queen Elizabeth Hospital, Birmingham, B15 2WB UK

**Keywords:** Burns, Cytokines, Genomics, Inflammation, Mortality, Sepsis, Trauma

## Abstract

As secondary complications remain a significant cause of morbidity and mortality amongst hospitalised trauma patients, the need to develop novel approaches by which to identify patients at risk of adverse outcome is becoming increasingly important. Centred on the idea that patients who experience “poor” outcome post trauma elicit a response to injury that is distinct from those who experience “good” outcome, tailored therapeutics is an emerging concept aimed at improving current treatment regimens by promoting patient-specific therapies. Making use of recent advancements in the fields of genomics, proteomics and metabolomics, numerous groups have undertaken a systems-based approach to analysing the acute immune and inflammatory response to major traumatic and thermal injury in an attempt to uncover a single or combination of biomarkers that can identify patients at risk of adverse outcome. Early results are encouraging, with all three approaches capable of discriminating patients with “good” outcome from those who develop nosocomial infections, sepsis and multiple organ failure, with differences apparent in blood samples acquired as early as 2 h post injury. In particular, genomic data is proving to be highly informative, identifying patients at risk of “poor” outcome with a higher degree of sensitivity and specificity than statistical models built upon data obtained from existing anatomical and physiological scoring systems. Here, focussing predominantly upon human-based research, we provide an overview of the findings of studies that have investigated the immune and inflammatory response to major traumatic and thermal injury at the genomic, protein and metabolite level, and consider both the diagnostic and prognostic potential of these approaches.

## Background

Despite recent advancements in the fields of fluid resuscitation and coagulopathy, traumatic injury remains the cause of more than five million deaths worldwide per year [1]. Moreover, those who survive the initial trauma are at an increased risk of multiple organ failure (MOF), acute respiratory distress syndrome (ARDS), nosocomial infections and sepsis, secondary complications that are associated with a range of adverse outcomes that include an extended length of intensive care unit (ICU) and hospital stay and an increased risk of death [2–6]. One factor thought to underlie these high mortality and morbidity rates is the trauma-induced alteration to immune and inflammatory responses. For instance, exaggerated immune and inflammatory reactions in the immediate aftermath of trauma are considered a contributory factor in the development of early MOF, whilst a state of immune paralysis, which develops in the days following injury, is viewed as a major factor underlying the increased susceptibility of trauma patients to hospital-acquired infections [7, 8].

In the settings of critical illness, traumatic brain injury (TBI) and blunt trauma, significant relationships have been reported between the immune response to injury and patient outcome. Trauma-induced alterations in the frequency, phenotype and/or function of circulating neutrophils, monocytes, natural killer cells and lymphocytes have been shown to be associated with patient mortality, increased length of hospital stay and the onset of such secondary complications as sepsis and ARDS (Table 1). However, there is little evidence to suggest that these associations are of any diagnostic or prognostic value. Thus, in recent years, with the aim of improving patient care, the emphasis of much trauma research has switched to biomarker discovery. Utilising the recent technological advancements in the fields of genomics, proteomics and metabolomics, several groups have performed detailed systems-based analysis of the immune and inflammatory response to major traumatic and thermal injury in an attempt to identify a single or combination of biomarkers that are of diagnostic and/or prognostic significance [9–13]. One of the long-term goals associated with this research is to reduce trauma-related morbidity and mortality rates via the implementation of patient-specific treatment and management protocols. Early results are encouraging, with genomic data providing information that in some instances has outperformed existing anatomical and physiological scoring systems in highlighting those trauma patients at risk of “poor” outcome [14, 15]. In this review, we summarise the findings of studies that have investigated the immune and inflammatory response to major traumatic and thermal injury at the genomic, protein and metabolite level, and consider the diagnostic and prognostic potential of these approaches. Given the differences that have been described in the immune/inflammatory response to severe injury between mice and humans [16], and the ongoing debate as to how closely murine models of trauma mimic the human response to injury [17–19], the findings discussed in this review, unless otherwise stated, have been generated from human-based studies.

**Table 1:** Trauma-induced changes in immunity that are associated with and/or predictive of adverse patient outcomes

		Increased risk of nosocomial infection	Development of sepsis	Mortality
Neutrophils	Frequency	Increased [60]		
	Phenotype	Decreased CD88 [80]	Increased CD11b [81] Decreased CD88 [80]	Decreased CD88 [80]
	Function	Decreased anti-microbial function [82]	Decreased anti-microbial function [83, 84] Decreased chemotaxis [85]	
Monocytes	Frequency	Decreased [60]		
	Phenotype	Decreased HLA-DR [80, 86]	Decreased HLA-DR [61, 87–89]	Decreased HLA-DR [88] Increased intracellular TLR9 expression [33]
	Function	Decreased LPS-induced TNF-α secretion [90, 91]		
Natural killer cells	Frequency	Decreased [92]		
Leukocytes	Frequency	Increased [60]		Increased [93]
	Gene expression	Increased expression of inflammation-related genes [15, 22]		Increased P38 MAPK and IL-6 expression [22, 23] Decreased expression of genes related to antigen presentation and T cell regulation [15, 22, 23]
Lymphocytes	Frequency	Decreased [60, 93]		Decreased [93]

*Abbreviations*: *HLA*-*DR* human leukocyte antigen-DR, *IL*-*6* interleukin-6, *LPS* lipopolysaccharide, *MAPK* mitogen-activated protein kinase, *TLR* toll-like receptor, *TNF* tumour necrosis factor alpha

## Review

### The genomic response to major traumatic injury

A technique that involves the mapping and sequencing of genes, genomics is a field of systems-based research that provides information on gene expression in a particular cell/tissue at any given time. Our current understanding of the genomic response to major traumatic injury is derived almost entirely from the findings of a large-scale collaborative research programme entitled “*Inflammation and the Host Response to Injury*”, in which genome-wide expression analysis has been performed on circulating leukocytes obtained from adults following either severe blunt trauma or thermal injury [14, 15, 20–24].

In a seminal paper published in 2011, Xiao and colleagues described altered expression of >80 % of the leukocyte genome following severe blunt trauma [22]. Referred to as a “genomic storm”, a total of 5136 genes exhibited a ≥two-fold change in expression relative to healthy controls, with pathway analysis revealing significant trauma-induced increases in the expression of genes involved in innate immunity, pathogen recognition and inflammation that occurred concomitant with reduced expression of genes related to antigen presentation and T cell function [22]. Time wise, whilst the greatest changes in gene expression were observed in samples obtained within 12 h of trauma, altered expression was still evident in leukocytes acquired 28 days post-injury, demonstrating that major blunt trauma is associated with both a rapid and prolonged genomic response [22].

Whilst clinical parameters such as injury severity score (ISS), blood transfusion and base deficit have little or no impact upon gene expression profiles post-trauma [22], patient age has been shown to strongly influence the genomic response to injury. Splitting a cohort of 244 severely-injured blunt trauma patients into young (<55 years) and old (≥55 years) sub-groups, Vanzant et al. reported significant age-associated differences in both the magnitude and duration of the genomic response to injury [24]. The group demonstrated that during the acute phase of injury (12–24 h), gene expression patterns of neutrophils isolated from younger adults were significantly more perturbed, relative to healthy controls, than those of older adults [24]. However, in the sub-acute period (day 4 post-injury), the reverse was observed, with the gene expression profiles of neutrophils from aged donors significantly more different from control subject values than those recorded for younger patients [24]. Thus, older adults elicit a unique genomic response to severe injury that is defined by an initial attenuated response that takes longer to return to a homeostatic baseline. Interestingly, with the exception of the work of Vanzant and colleagues [24], all studies published to date that have investigated the genomic response to trauma have analysed samples collected from patients aged 16–55 years [14, 15, 20–23]. Thus, it is currently unclear as to what ramifications, if any, the data of Vanzant et al. [24] will have for the proposed use of genomics as a prognostic indicator of patient outcome. It may be that separate cohorts of healthy young and older adults are needed in order to serve as age specific reference ranges to which the genomic profiles of traumatically injured young and geriatric patients would be compared. Alternatively, an approach in which patients serve as their own internal controls for gene expression changes could be adopted. This strategy of studying “within-patient gene expression changes” (WPEC), which involves quantifying per-hour log-fold changes in gene expression in the post-injury phase, has proven successful in a cohort of blunt trauma patients, where WPEC were strongly associated with long-term post-injury complications [23].

#### The genomic response to major trauma and its relationship with patient outcome

Several studies have shown that in the immediate hours and days following major traumatic injury, gene expression profiles of circulating leukocytes differ between patients who experience “good” outcome and those who report “poor” outcome. Interestingly, it is the magnitude and duration of the genomic response rather than the directional changes in gene expression that discriminate these two groups from one another [14, 20, 22]. Indeed, relative to healthy controls, both groups elicit a genomic response that is characterised by the up-regulation of genes involved in cytokine production and the synthesis/degradation of inflammatory lipid mediators, and the down-regulation of genes related to antigen presentation and T cell function [20, 22]. However, patients with complicated recovery (defined as recovery >14 days, no recovery or death) display a unique kinetic profile that is defined by more robust early changes (<12 h post-injury) in gene expression that fail to return to a homeostatic baseline as quickly as that observed in patients who report “good” outcome [20, 22]. Moreover, the further the genomic profile of a patient differs from that of a control, the more likely they are to develop nosocomial infections and experience longer lengths of both ICU and hospital stay [21]. Importantly, these associations remained after controlling for the effects of injury severity and physiological dysfunction [21].

Data are beginning to emerge suggesting that early genomic profiling may have potential as a highly sensitive prognostic tool for the identification of trauma patients at risk of adverse outcome. In a cohort of 96 severely-injured blunt trauma patients, Cuenca et al. demonstrated that an assessment of leukocyte gene expression within 24 h of injury could differentiate patients who experienced a complicated recovery from those with an “uneventful” outcome, with an area under the receiver operator curve (AUROC) value of 0.811 [14]. Similarly, in a group of thermally-injured patients, genomic scoring correctly predicted the development of multiple hospital-acquired infections in 80 % of patients [15]. Interestingly, in both studies, models built on genomic analysis outperformed the predictive capabilities of models based on clinical, anatomical and physiological data [14, 15], suggesting that genomic analysis provides unique information on a patients response to traumatic injury that supersedes the prognostic capacity of current scoring systems.

Given that >80 % of the leukocyte transcriptome is altered post-trauma, changes in a single or subset of genes are unlikely to emerge as biomarkers for the identification of patients at risk of poor outcome [22]. Rather, it will be information derived from the assessment of changes in global gene expression that will form the basis of any future prognostic models developed on the back of genomic data. On this note, mathematical systems have been developed that can simplify changes that occur in gene expression across the entire genome into a single number. This approach has been applied to trauma studies and proven successful in identifying those patients who experience “poor” outcome [14, 21]. However, the major drawback of these studies as well as those described above is that all genomic data has been derived from and tested within patients recruited as part of a single trauma study, namely the *Inflammation and the Host Response to Injury* programme [14, 15, 20–24]. Thus, until these data and the associated scoring systems have been tested and validated in independent cohorts of trauma patients, its suitability as a prognostic indicator of patient outcome should be viewed with caution [25].

#### The prognostic value of mitochondrial DNA

**Fig. 1:**
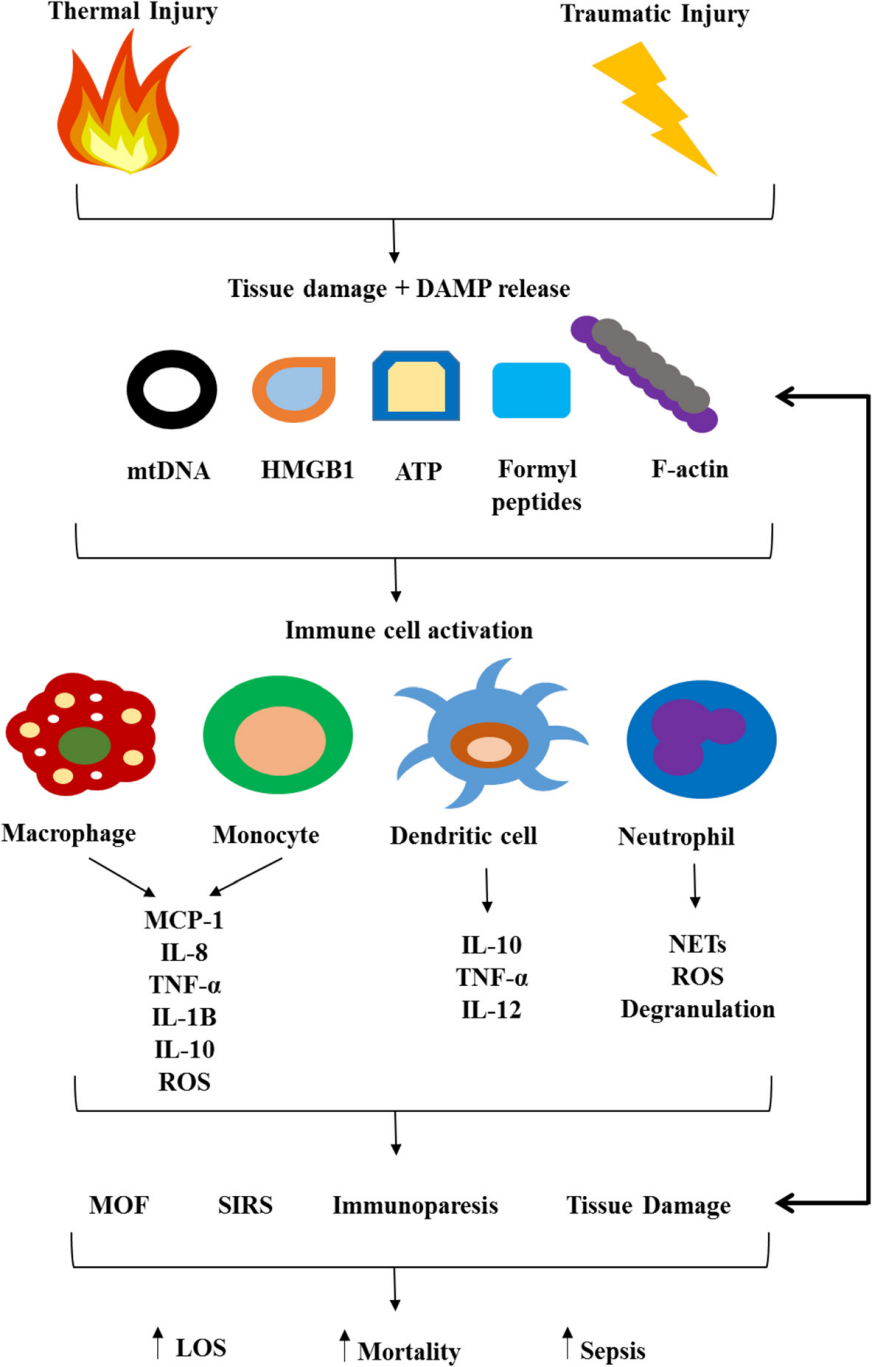
Damage Associated Molecular Patterns (DAMPs) release and immune cell activation following sterile traumatic injury. Tissue damage arising from traumatic or thermal injury results in the release into circulation of mitochondrial (e.g. mtDNA, formyl peptides), cytosolic (e.g. F-actin) and nuclear (e.g. HMGB1)-derived damage associated molecular patterns (DAMPs). Through binding to pathogen recognition receptors, DAMPs trigger the activation of circulating immune cells resulting in the secretion of pro- and anti-inflammatory cytokines as well as a series of functional responses, which include the generation of reactive oxygen species (ROS) and neutrophil extracellular traps (NETs). Together, cell activation and cytokine secretion creates an inflammatory environment that favours the development of multiple organ failure, tissue damage and immunoparesis, conditions that are associated with a range of poor patient outcomes, which include a longer length of hospital stay and an increased risk of sepsis and mortality. Tissue damage arising from immune cell activation would lead to the release of further DAMPs, creating a vicious cycle, with continued inflammation and immune activation. *ATP* adenosine tri phosphate, *DAMP* damage-associated molecular pattern, *F*-*actin* filamentous actin, *HMGB1* high-mobility group box 1 protein, *IL* interleukin, *LOS* length of stay, *MCP*-*1* monocyte chemoattractant protein 1, *MOF* multiple organ failure, *mtDNA* mitochondrial DNA, *NETs* neutrophil extracellular traps, *ROS* reactive oxygen species, *SIRS* systemic inflammatory response syndrome, *TNF*-*α* tumour necrosis factor-alpha

The systemic inflammatory response syndrome (SIRS) that occurs in the immediate aftermath of major traumatic injury is thought to be triggered in part by the release from damaged, stressed or necrotic tissue of damage associated molecular patterns (DAMPs). A collection of cytosolic, nuclear and mitochondrial-derived proteins and DNA, DAMPs activate circulating immune cells via interaction with pathogen recognition receptors (PRRs), which include members of the toll-like receptor (TLR) family [26] (Fig. 1).

DAMP release has been proposed as a mechanistic explanation for the near identical gene expression profiles reported for leukocytes isolated from severe blunt and thermally-injured patients [22]. Although markedly different in their mechanism of injury, it has been suggested that both blunt and thermal trauma leads to the release into the circulation of DAMPs, whose subsequent PRR-mediated activation of leukocytes triggers a common genomic response [22]. Aside from genomics, we have recently shown mitochondria-derived DAMPs to be potent inducers of interleukin (IL)-8 secretion by human neutrophils in vitro [27], whilst others have demonstrated in vivo administration of mitochondrial DNA (mtDNA) triggers an inflammatory response in rats that resembles many facets of the SIRS response observed in traumatically-injured humans [28, 29]. Thus, if capable of influencing both the genomic and inflammatory response to injury, could a measurement of circulating DAMPs provide information that is of prognostic significance?

In TBI subjects [30] and cohorts of mixed trauma patients [31, 32], a measurement of circulating mtDNA levels at hospital admission or in the days following injury has identified patients at risk of poor outcome. Significantly higher levels of mtDNA have been reported in plasma and cerebrospinal fluid samples from subjects who subsequently developed SIRS/MOF during their hospital stay or who exhibited poor functional outcome at follow up [30, 31]. Furthermore and of particular interest, three studies have demonstrated a relationship between mtDNA and patient survival, with the levels of this DAMP significantly elevated in the samples of non-survivors compared to survivors [30, 32, 33]. In terms of potential clinical relevance, inclusion of mtDNA levels into a scoring system that contained information on patient physiology and gender was shown to significantly improve the power of this model in discriminating survivors from non-survivors, suggesting that an early assessment of mtDNA levels may be of prognostic benefit [33].

Rich in unmethylated CpG motifs, mtDNA is recognised by the PRR TLR9 [29]. In a prospective observational study of critically-ill patients, Krychtiuk et al. found no significant difference in the intracellular expression levels of TLR9 in monocytes between survivors and non-survivors [33]. However, when analysed in the context of mtDNA levels, TLR9 was found to be a significant predictor of survival in patients with “high” but not “low” plasma levels of mtDNA [33]. Related to this, the group also showed mtDNA was only significantly associated with mortality in those patients they defined as possessing “high” intracellular levels of TLR9 [33]. Thus, unsurprisingly, patients stratified as “high” for both plasma mtDNA and TLR9 expression exhibited the greatest risk of death within 30 days of hospital admission [33]. Mechanistically, this relationship between mtDNA, TLR9 and mortality has been proposed to reflect a mtDNA-driven hyper activation of the innate immune system [33].

Taken together, these data suggest that either in isolation or in combination with other clinical assessments, a measure of mtDNA levels and its receptor TLR9 in the early phase post-injury provides information that has the potential to assist in the identification of patients at increased risk of poor outcome.

#### MicroRNA screening as a tool for the early diagnosis and stratification of traumatic injury

MicroRNAs (miRNAs) are a highly conserved class of short single-stranded non-coding RNA molecules, which via translational repression or degradation of messenger RNA play an important role in regulating gene expression [34]. In rodents [35–37] and humans [38, 39], traumatic injury leads to significant alterations in the circulating levels of miRNAs. For instance, when compared to healthy controls, the levels of circulating miRNAs predicted to regulate the expression of pro- and anti-inflammatory cytokine genes (e.g., miR-125a, miR-181, miR-202, miR-374b and miR454) as well as genes involved in neuronal survival and central nervous system signalling have been shown to be increased or decreased in samples obtained post-polytrauma or TBI [36, 38].

On current evidence, miRNA screening appears to hold promise as a tool for both the diagnosis of TBI and the stratification of these patients according to injury severity [35, 36, 39]. Compared to the values of healthy subjects, decreased levels of miR-16 and miR-92a, and increased levels of miR-765 have been reported in plasma samples acquired from severe TBI patients 24–48 h post-injury [39]. Whilst as single entities, miR-16, miR-92a and miR-765 were considered good markers of severe TBI (AUROC values of 0.89, 0.82 and 0.86 respectively), in combination these miRNAs discriminated with 100 % sensitivity and 100 % specificity severe TBI patients from controls [39]. Similarly, significant differences have been found in the serum levels of 13 miRNAs between sham mice and those subjected to differing degrees of mild TBI (mTBI) [36]. Given the lack of immediate symptoms associated with mTBI, miRNA profiling may be a useful tool by which to improve upon the current methods of diagnosis, which revolve around the interpretation of imaging data and clinical examination [36].

Aside from the diagnosis of TBI, miRNA profiling may assist in the identification of those trauma patients at an increased risk of hospital-acquired infection. Measuring circulating miRNA levels in samples acquired from 30 polytrauma patients upon hospital admission and 24 h post-injury, Owen et al. found significantly lower levels of miR-125a and miR-374b in samples from patients who subsequently developed pneumonia [38]. Predicted to target IL-10 mRNA, it was suggested that decreased levels of miR-125a and miR-374b could increase patient susceptibility to infection by creating an immune suppressive environment [38]. On this note, in a larger cohort of polytrauma patients, the group had previously reported elevated IL-10 mRNA levels at 24 h post-injury were associated with the development of bacteraemic episodes [40].

### The inflammatory response to major traumatic injury

Two distinct responses characterise the immune and inflammatory reactions to major traumatic injury; a systemic inflammatory response, defined by elevated levels of circulating pro-inflammatory cytokines and immune cell activation, and a compensatory anti-inflammatory response characterised by increased levels of anti-inflammatory cytokines and immunoparesis [41]. For many years, it was thought that these two responses were sequential. However, at both the transcript [22] and protein [10, 42, 43] levels, data has recently emerged that challenges this long-standing paradigm, with evidence of a simultaneous increase in pro- and anti-inflammatory responses post-trauma. Indeed, across a range of trauma cohorts, elevated levels of pro- and anti-inflammatory cytokines have been measured in samples obtained 2–24 h post-injury [10, 42, 43]. Faced with these data, numerous groups have investigated whether patients that elicit a more robust inflammatory response to trauma are at an increased risk of adverse outcome (Table 2).

**Table 2:** Examples of trauma-induced inflammatory responses that are associated with and/or predictive of adverse patient outcomes

	Increased risk of nosocomial infection	Development of sepsis	Development of MODS/MOF	Mortality
IL-6	Elevated levels at admission post blunt trauma [60]	Elevated levels following thermal injury [55, 56]	Elevated levels post severe trauma [43, 53, 94]	Elevated in non-survivors following thermal injury [10, 11, 45, 47] and TBI [42]
IL-8		Elevated levels following thermal injury [55, 56]		Elevated in non-survivors following thermal injury [10, 45, 47] and TBI [42]
IL-10			Elevated levels post severe trauma [43]	Elevated levels associated with increased risk of death post thermal injury [95] and TBI [42, 50]
IL1-RA	Elevated levels at admission post blunt trauma [60]		Elevated levels post severe traum [43, 94]	Elevated in non-survivors following thermal injury [11, 47]
MCP-1	Elevated levels at admission post blunt trauma [60]			Elevated in non-survivors following thermal injury [10, 11, 47]

*Abbreviations*: *IL* interleukin, *IL1*-*RA* interleukin 1 receptor antagonist, *MCP*-*1* monocyte chemoattractant protein, *MODS* multiple organ dysfunction syndrome, *MOF* multiple organ failure, *TBI* traumatic brain injury

#### Cytokines and their relationship with patient outcome

##### Mortality and MOF

In burns research, several studies have shown that a measure of circulating inflammatory cytokines and chemokines in the early post-injury phase can distinguish survivors from non-survivors. In cohorts of paediatric and adult patients, elevated levels of interleukin IL-6, IL-8, IL-10, IL-15, monocyte chemoattractant protein-1 (MCP-1), granulocyte-colony stimulating factor (GCSF), IL-1 receptor antagonist (IL1-RA) and eotaxin as well as reduced levels of IL-4, IL-7 and IL-13 have been detected in plasma/serum samples of non-survivors compared to survivors [10, 11, 44–47]. These differences, which are evident in samples acquired at hospital admission and/or days three and seven post-trauma, suggest that an immediate assessment of the inflammatory response to thermal injury may have prognostic value. Importantly, elevated levels of cytokines remain significantly associated with mortality after controlling for factors known to increase the risk of death post-burn injury such as patient age, percent total body surface area (TBSA) burned and inhalation injury [45, 47]. Furthermore, and of particular interest, Finnerty et al. showed in a cohort of 330 paediatric patients with >25 % TBSA burn that combining measurements of circulating cytokines with clinical information improved the predictive accuracy of models built on clinical variables alone by 29 %, highlighting the benefit that the inclusion of laboratory-derived data can have on current medical indicators of patient outcome [46].

Akin to thermal injury, circulating concentrations of inflammatory cytokines have been found to differ between survivors and non-survivors of TBI and polytrauma, with elevated levels of IL-6, IL-8 and/or IL-10 present in samples obtained within 24 h of injury from patients who succumb to their injuries [42, 48–50]. Of note, in a cohort of 93 patients with TBI, Soares et al. found that within 10 h post-injury, patients with high serum IL-10 levels (>90 pg/ml) were six times more likely to die than those with low IL-10 levels (<50 pg/ml) independent of ISS and age [50]. Relationships between raised serum cytokines and poor outcome post-TBI may be related in part to the role of these inflammatory mediators in the development of secondary complications [51].

Multiple organ dysfunction syndrome (MODS) and MOF are major secondary complications amongst hospitalised trauma patients that are associated with increased mortality rates and length of hospital stay [43]. A measure of circulating inflammatory cytokines in the early hours/days post-injury has been shown to be capable of identifying those patients at an increased risk of MOF. Following, major chest trauma [43], TBI [52], polytrauma [49] and severe injury [53], significantly elevated levels of multiple cytokines and chemokines such as IL-1RA, IL-6 and IL-10 have been recorded in samples obtained from those patients who subsequently develop MOF. Of these studies, Cuschieri and co-workers demonstrated that a plasma concentration of IL-6 ≤ 350 pg/ml within 12 h of severe blunt trauma could identify with 88 % accuracy those patients who would not develop MODS during their hospital stay [53].

##### Nosocomial infection and sepsis

Nosocomial infections and sepsis are common secondary complications amongst hospitalised trauma patients. In thermally-injured subjects in particular, sepsis is not only a highly prevalent complication but a significant cause of mortality, with sepsis-related deaths in this cohort exceeding those reported in the settings of traumatic injury and critical care [54]. Within 24 h of burn injury, IL-6 and IL-8 levels have been found to be significantly greater in serum samples obtained from patients that subsequently develop sepsis during their hospital stay when compared to those who do not [55–58]. Furthermore, a measure of inflammatory cytokine levels during septic episodes can provide information on infection-related outcome. In a study of 60 burn-injured patients, Pileri and colleagues demonstrated that a cut-off value for IL-10 of 60 pg/ml at day three post-injury could discriminate septic survivors from non-survivors with 93 % specificity and 92 % sensitivity [57].

In addition to thermally-injured, a measure of the inflammatory response following TBI and blunt trauma can identify patients at risk of sepsis/nosocomial infection [59, 60]. Interestingly, as reported in genomic studies [14, 20, 22], it is the magnitude of the inflammatory response that discriminates infected patients from their non-infected counterparts, with the former group eliciting a more robust inflammatory response in the immediate aftermath of injury [60].

Despite numerous studies reporting associations between circulating cytokine levels and infection, the use of cytokine measurements as a stand-alone tool for the early identification of sepsis is hampered by insufficient sensitivity and specificity. Interestingly, in a recent prospective observational study of 100 severely-injured trauma patients, Cheron et al. demonstrated that combining an assessment of a patient’s immune status with a measurement of circulating IL-6 concentrations could improve both the specificity and positive predictive value of models built on cytokine data alone [61]. Thus, this result highlights the potential of combining different systems-based approaches for the development of more accurate and reliable models for use in the diagnosis and/or prognosis of sepsis amongst hospitalised trauma patients.

### The metabolomic response to major traumatic injury

A technique that identifies and quantifies metabolites within biological fluids, cells and tissues, metabolomics is a systems-based approach for the profiling and analysis of cellular processes. A long-standing interest of researchers in the fields of inflammatory disease and infection [62–67] metabolic profiling is an emerging area of research in the settings of critical care and trauma. In animal models of polytrauma with haemorrhagic shock [68–73], as well as in cohorts of TBI [74], burns [75] and major trauma [9, 12, 76, 77] patients, analysis of blood [9], plasma [9, 12, 72, 73, 75, 76], serum [69, 71, 74], lymph [77] and urine [70, 71] collected in the hours and days post-injury has shown severe metabolic disruption to be a consequence of trauma. For example, marked alterations, relative to healthy controls, have been reported in the concentrations of multiple metabolites such as serine, lactate, succinate, carnitine and citrate, demonstrating that trauma leads to disturbances in carbohydrate, protein and fatty acid metabolism [9, 12, 68–77].

Besides simply describing the changes that occur in the circulating “metabolome” post-injury, a handful of groups have investigated whether metabolic profiling has the potential to serve as a prognostic tool. In one of the first studies to examine the relationship between trauma-induced metabolic derangement and patient outcome, Cohen et al. found in a cohort of major trauma patients that survivors and non-survivors could be clearly discriminated by the concentrations of triacylglycerol, phospholipids and monounsaturated fatty acids in admission blood samples, with non-survivors presenting with significantly lower levels of all three lipid metabolites [9]. Similar to these observations, Lexcen and co-workers recently reported in a porcine model of polytrauma and haemorrhagic shock that when compared to survivors, concentrations of succinate and O-phosphocholine were significantly increased and decreased, respectively, in animals that succumbed to their injuries [69]. Aside from mortality, metabolic profiling in the early post-injury phase may be a useful approach for identifying patients that are at an increased risk of post traumatic complications [74, 78, 79]. For example, in a cohort of 22 severely-injured patients, a metabolomic assessment of plasma samples acquired at admission to ICU was found to identify with a reasonable degree of certainty (AUROC = 0.778) patients that subsequently developed sepsis during their hospital stay [78]. Interestingly, in an independent cohort of ICU patients, Mickiewicz et al. recently demonstrated that a combination of metabolomic and proteomic data could accurately discriminate, with a sensitivity of 0.94 and a specificity of 1, septic shock patients from those undergoing a SIRS response in the absence of infection [79]. Shown to perform better than statistical models built on clinical scoring systems, these data highlight the improved discriminatory power that can be gained by combining system-based approaches [79].

Although in its infancy, metabolic profiling of trauma patients has potential prognostic utility for identifying individuals that are at an increased risk of poor outcome. However, given the limited number of studies and the small size of the patients cohorts analysed, further work is needed to not only validate published observations but also address whether clinical (e.g. ISS) and patient (e.g. gender, age) variables influence the metabolomic response to injury. Furthermore, in the context of tailored therapeutics, it will be interesting for future studies to examine whether the circulating “metabolome” can provide information on a patients response to treatment regimens (e.g. resuscitation) or therapeutic interventions, which if proven to be the case, could lead to the implementation of patient-specific management protocols.

## Conclusions

Although in its infancy, it is evident that a system-based approach to studying the immune and inflammatory response to severe traumatic and thermal injury has the potential to generate data that can influence patient care. Once laborious and time-consuming, recent technological advancements have revolutionised the practicalities of systems-based approaches, making it possible for instance to perform genome-wide expression analysis within 12–18 h [14]. By identifying with a high degree of accuracy patients at risk of “poor” outcome, system-based approaches have in some instances outperformed the prognostic capacity of existing clinical scoring systems [14, 15], meaning it is conceivable that metabolomic, proteomic and/or genomic data will be utilised in the future to assist in the creation of tailored treatment and/or management protocols. Interestingly, across multiple cohorts of patients with differing mechanisms of injury, alterations in innate immune responses, inflammatory pathways and adaptive immunity have consistently been linked to adverse outcome, suggesting that a common mechanism may underlie the dysregulated immune and inflammatory response of patients that experience high morbidity and mortality rates post-injury. Investigating such mechanisms should be the focus of futures studies, with the long-term goal of identifying novel therapeutic targets.

## Abbreviations

ARDS: Acute respiratory distress syndrome
AUROC: Area under the receiver operator curve
DAMPs: Damage-associated molecular patterns
GCSF: Granulocyte-colony stimulating factor
HLA-DR: Human leukocyte antigen-DR
HMGB-1: High-mobility group box-1
ICU: Intensive care unit
IL: Interleukin
IL1-RA: Interleukin-1 receptor antagonist
ISS: Injury severity score
LPS: Lipopolysaccharide
MAPK: Mitogen-activated protein kinase
MCP-1: Monocyte chemoattractant protein-1
miRNA: MicroRNA
MODS: Multiple organ dysfunction syndrome
MOF: Multiple organ failure
mTBI: Mild traumatic brain injury
mtDNA: Mitochondrial DNA
PRR: Pathogen recognition receptor
SIRS: Systemic inflammatory response syndrome
TBI: Traumatic brain injury
TBSA: Total body surface area
TLR: Toll-like receptor
TNF-α: Tumour necrosis factor alpha
WPEC: Within-patient gene expression changes
